# NcRNA-regulated CAPZA1 associated with prognostic and immunological effects across lung adenocarcinoma

**DOI:** 10.3389/fonc.2022.1025192

**Published:** 2023-01-04

**Authors:** Tingting Qin, Wanping Xiang, Yiming Mao, Hongyan Zhai, Zhihao Yang, Hongpan Zhang

**Affiliations:** ^1^ Department of Oncology, Wuhan Third Hospital, Wuhan, Hubei, China; ^2^ North Sichuan Medical College, Nanchong, Sichuan, China; ^3^ Department of Thoracic Surgery, Affiliated Hospital of North Sichuan Medical College, Nanchong, Sichuan, China; ^4^ Suzhou Kowloon Hospital, Shanghai Jiao Tong University School of Medicine, Suzhou, Jiangsu, China; ^5^ Department of Oncology, Linfen People’s Hospital, Linfen, Shanxi, China; ^6^ Tianjin Key Laboratory of Medical Epigenetics, Key Laboratory of Breast Cancer Prevention and Therapy (Ministry of Education), Department of Biochemistry and Molecular Biology, Tianjin Medical University, Tianjin, China; ^7^ Department of Oncology, Affiliated Hospital of North Sichuan Medical College, Nanchong, Sichuan, China

**Keywords:** LUAD, CAPZA1, ceRNA, prognosis, immunotherapy

## Abstract

Recent discoveries have suggested that the F-actin capping protein α1 subunit (CAPZA1) in various human tumors could play a significantly important role in regulating cell proliferation, metastasis, and epithelial–mesenchymal transition. However, the immune-regulating role of CAPZA1 in the initiation and development of lung adenocarcinoma (LUAD) remains unclear. In our research, we first found that CAPZA1 serves as an oncogene in pan-cancers from the TCGA data and higher CAPZA1 expression process unfavorably prognostic value in LUAD based on starBase database, PrognoScan, and LOGpc database. Then, in our analyses, lncRNAs AC026356.1 in LUAD acted as a competitive endogenous RNA (ceRNA) of miR-30d-5p, which might be the possible regulatory miRNA of CAPZA1 based on the starBase database. Finally, we confirmed that CAPZA1 expression had a tightly positive correlation with immune infiltration cells, immune infiltration markers, TMB, MSI, immune score, stromal score, and immune checkpoints, indicating that CAPZA1 was a markedly reliable therapeutic target for immunological antitumor strategies. In conclusion, our investigations revealed that CAPZA1 might function as an immune-associated biomarker in the development and treatment of LUAD, thereby acting as a promising prognostic and therapeutic target against LUAD.

## 1 Introduction

Lung cancer (LC) has gradually become a leading cause of cancer-related morbidity and mortality worldwide among men and women (1, 2). Lung adenocarcinoma (LUAD) accounts for over 40% of lung cancer, with the most common subtype being lung cancer (3). Despite advances in therapeutic strategies such as surgical techniques, radiotherapy, and chemotherapy, the survival of patients with LUAD is still unfavorable (4). Immunotherapy has been a promising and effective treatment strategy for multiple cancers (5–7), including LUAD (8). However, there are some limitations regarding immune checkpoint inhibitors in this study: Over half of the LUAD patients are not markedly sensitive to PD-1/PD-L1 antitumor immunotherapy, indicating the existence of additional immunotherapeutic biomarkers in the LUAD tumor microenvironment (9). Therefore, we propose identifying immune-associated prognostic biomarkers to conquer LUAD for timely detection, early diagnosis, prognosis, and treatment.

F-actin capping protein α1 subunit (CAPZA1) belongs to the capping protein (CP), which is a family of the actin filament (F-actin) that is a critical component of the cytoskeleton, and the dynamic remodeling of these filaments provides the impetus for cell invasion and migration (10–13). Recent discoveries have suggested that CAPZA1 in various human tumors could regulate cell proliferation, metastasis, and epithelial–mesenchymal transition (14, 15), and elevated CAPZA1 is closely linked to an unfavorable prognosis in gastric cancer (16). For instance, PIM1 phosphorylated CAPZA1 and was responsible for increasing prostate cancer adhesion and migration *in vitro* (17). Additionally, during the immune process, the actin cytoskeleton plays a pivotal role in controlling B-cell antigen uptake, polarization, presentation, T-lymphocyte activation, and effector functions in the tumor microenvironment (TEM) (18, 19). To the best of our knowledge, the immune-regulating role of CAPZA1 in LUAD development remains incompletely understood.

This current study investigated the expression profiling and prognostic value of CAPZA1 in pan-cancers based on several databases. Then, ncRNAs, containing miRNAs and lncRNAs, participated in the regulation of CAPZA1 in LUAD. High CAPZA1 levels in LUAD were also associated with immune cell infiltration and immune checkpoints, suggesting that ncRNAs-mediated upregulation of CAPZA1 is an immune-related biomarker with a worse impact on clinical outcomes.

## 2 Material and methods

### 2.1 Data collection and processing

The mRNA expression data and somatic mutation data of the LUAD in The Cancer Genome Atlas (TCGA) database were acquired using UCSC Xena (https://xena.ucsc.edu/) (20). MSI data was downloaded from the research of Russell Bonneville (21). The flow chart is shown in **Supplementary Figure 1**.

### 2.2 StarBase online database

The starBase database (http://starbase.sysu.edu.cn/) (22) was employed to identify differentially expressed CAPZA1 and its prognostic value in many cancers. The expression of CAPZA1 is presented by FPKM (fragments per kilobase of transcript sequence per million base pairs sequenced). Firstly, the starBase database involving PITA, RNA22, miRmap, microT, miRanda, PicTar, and TargetScan was employed to predict potential miRNAs that directly bond to CAPZA1 upstream in LUAD. This was done to speculate about their differential expression and OS. Next, we predicted 16 miRNAs, of which six were negatively correlated with CAPZA1 expression, so we chose the one least correlated, mirRNA-30d-5p. Then, we forecast potential hsa-miR-30d-5p-modulated lncRNAs in LUAD and analyzed the differential expression level and prognosis of the miRNAs screened. There were two lncRNAs that met the requirements for screening eight lncRNAs. Finally, the starBase database was also used to examine the miR-30d-5p-CAPZA1, miR-30d-5p-AC026356.1, and AC026356.1-CAPZA1 interactions. *P <*0.05 was treated as the significance threshold for all statistical analyses.

### 2.3 Survival analysis

The CAPZA1 survival analysis of CAPZA1 expression in LUAD was validated through the PrognoScan database (http://www.abren.net/PrognoScan/) (23) and LOGpc (Long-term Outcome and Gene Expression Profiling Database of pan-cancers, https://bioinfo.henu.edu.cn/DatabaseList.jsp). A corrected P-value <0.05 was regarded as a statistically significant difference.

### 2.4 Immune cell infiltration and immune checkpoint analysis

Additionally, we applied the TIMER online database (https://cistrome.shinyapps.io/timer/) (24), a comprehensive resource for systematic analysis of tumor immune infiltrates, immune infiltrate biomarkers, and immune systems checkpoint across LUAD into CAPZA1-associated the abundance of the immune response. The immunophenoscore (IPS), which can be obtained from the TCIA website (https://tcia.at/home), has been verified to predict patients’ response to immune checkpoint inhibitors (ICIs). A higher IPS has a better outcome with ICI treatment (25).

### 2.5 Correlation between CAPZA1 and immune characteristics

Recent studies have shown that tumor mutational burden (TMB) predicts the response to the clinical efficacy of anti-PD1/PD-L1 treatment (26). Microsatellite instability (MSI) (27) has also been considered an indispensable biomarker for TME. To further evaluate CAPZA1 in LUAD immunity, we examined the link between CAPZA1 expression and MSI and TMB. Finally, ImmuneScore and StromalScore analyses were carried out *via* the “estimate” R package.

### 2.6 Gene set enrichment analysis

GSEA (28) (version 3.0, http://www.broadinstitute.org/gsea/index.jsp) has been utilized for evaluating gene sets or biological pathways, which varied significantly between CAPZA1 in high- and low-expression level groups. In this study, the following settings were used: 500 was the maximum size of the gene set, while 15 was the minimum size of the gene set, with 1,000 as the number of permutations, along with improved gene sets with an FDR (false discovery rate) q-val <0.05 having been measured as significant.

## 3 Results

### 3.1 CAPZA1 mRNA expression in pan-cancers

**Figure 1:**
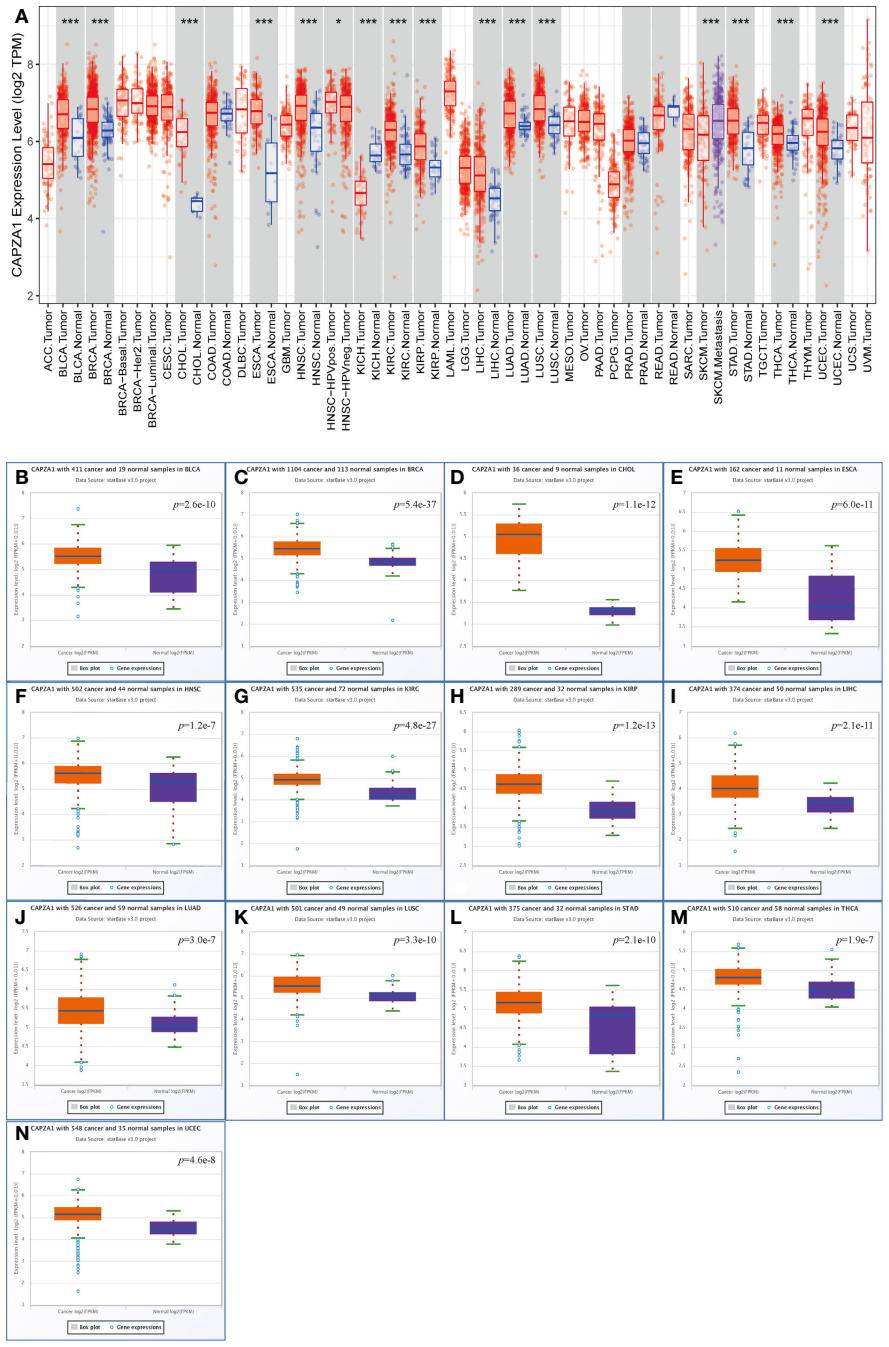
Expression analysis for CAPZA1 in multiple cancers. **(A)** The expression of CAPZA1 in 33 types of human cancer based on the TCGA cancer and normal data in TIMER. **(B–N)** CAPZA1 expression in the starBase BLCA. **(B)**, BRCA **(C)**, CHOL **(D)**, ESCA **(E)**, HNSC **(F)**, KIRC **(G)**, KIRP **(H)**, LIHC **(I)**, LUAD **(J)**, LUSC **(K)**, STAD **(L)**, THCA **(M)**, and UCEC **(N)** tissues compared with corresponding TCGA normal tissues. *p-value < 0.05; ***p-value < 0.001.

CAPZA1 mRNA levels from 33 cancer types in the TCGA were applied to TIMER. The results indicated that the expression of CAPZA1 was relatively high in 13 cancers, such as BLCA, BRCA, CHOL, ESCA, HNSC, KIRC, KIRP, LIHC, LUAD, LUSC, STAD, THCA, and UCEC, while the deletion of CAPZA1 was in KICH and SKCM (**Figure 1A**). We further utilized the starBase database to verify whether CAPZA1 expression is overexpressed in multiple tumor tissues. **Figures 1B–N** showed that CAPZA1 mRNA expression in the 13 types were still upregulated.

### 3.2 Prognostic potential of CAPZA1 in pan-cancers

**Figure 2:**
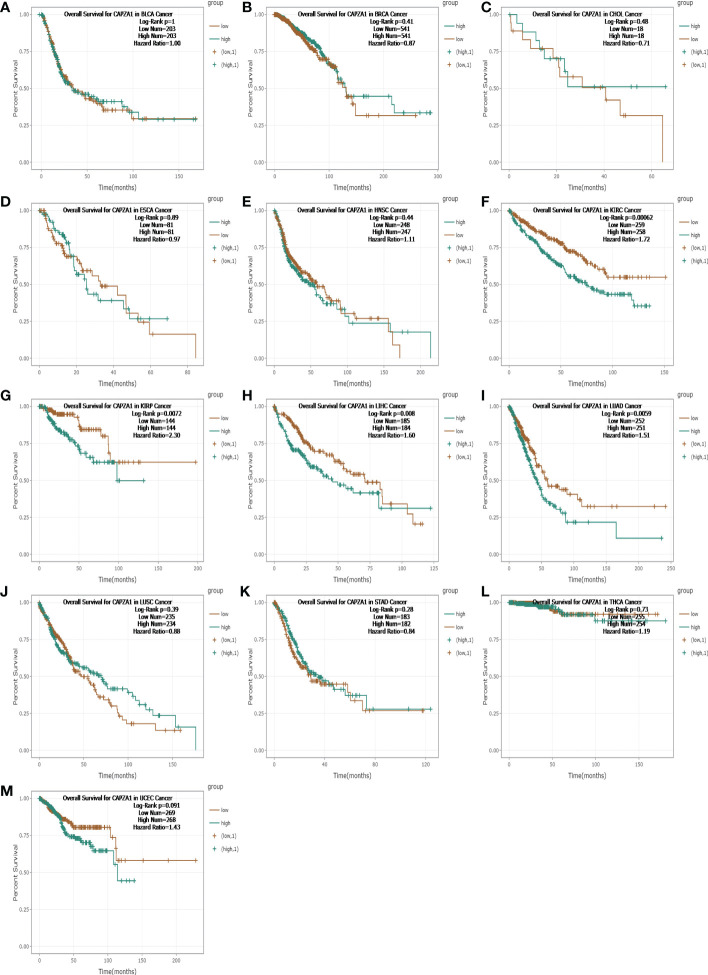
The overall survival (OS) analysis for CAPZA1 in various human cancers determined by starBase. **(A–M)** The OS plot of CAPZA1 in BLCA. **(A)**, BRCA **(B)**, CHOL **(C)**, ESCA **(D)**, HNSC **(E)**, KIRC **(F)**, KIRP **(G)**, LIHC **(H)**, LUAD **(I)**, LUSC **(J)**, STAD **(K)**, THCA **(L)**, and UCEC **(K)**.

**Figure 3:**
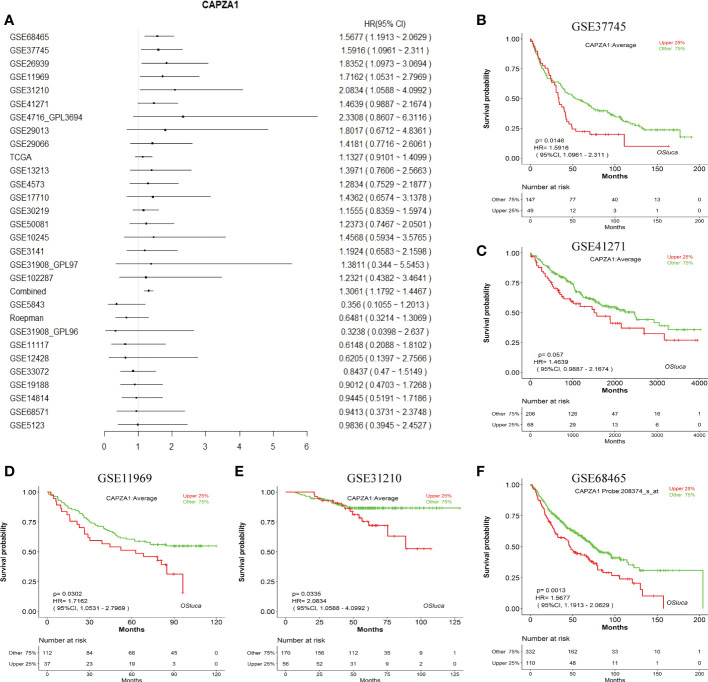
The prognostic value of CAPZA1. **(A)** Prognostic value of CAPZA1 in multiple data sets. **(B–F)** The representative survival curves.

Firstly, the starBase database was employed to explore the prognosis of CAPZA1 in pan-cancer data downloaded from the TCGA. The result indicated that CAPZA1 at high expression afforded worse OS in KIRC, KIRP, LIHC, and LUAD (**Figures 2A–M**). Then, the fact that patients with high CAPZA1 expression had an unfavorable long-term prognosis in LUAD was also validated in PrognoScan (**Table 1**) and the LOGpc database (**Figures 3A–F**). These findings indicated that overexpressed CAPZA1 might be a worse prognostic marker in LUAD.

**Table 1:** The Prognostic value of CAPZA1 in lung adenocarcinoma in PrognoScan.

GENE_NAME	DATASET	CANCER TYPE	ENDPOINT	N	COX P-VALUE	ln(HR)	HR [95% CI-low CI-upp]
CAPZA1	GSE31210	Adenocarcinoma	Relapse Free Survival	204	8.27E-05	3.90283	49.54 [7.10 - 345.90]
CAPZA1	GSE13213	Adenocarcinoma	Overall Survival	117	0.000201	0.742359	2.10 [1.42 - 3.11]
CAPZA1	jacob-00182-MSK	Adenocarcinoma	Overall Survival	104	0.06556	2.05522	7.81 [0.88 - 69.60]
CAPZA1	GSE31210	Adenocarcinoma	Overall Survival	204	0.001298	4.45077	85.69 [5.69 - 1290.75]
CAPZA1	GSE13213	Adenocarcinoma	Overall Survival	117	0.03585	0.534665	1.71 [1.04 - 2.81]

### 3.3 Prediction and analysis of upstream miRNAs of CAPZA1

**Figure 4:**
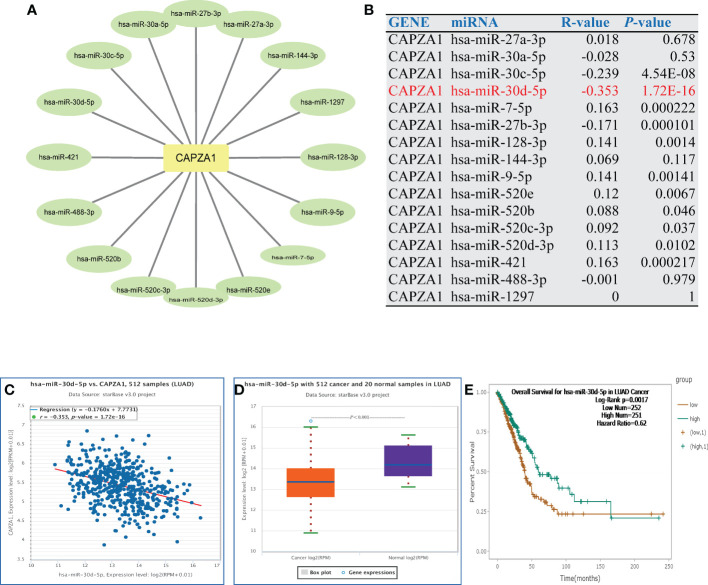
Identification of miR-30d-5p as a potential upstream miRNA of CAPZA1 in LUAD. **(A)** The miRNA- CAPZA1 regulatory network established by Cytoscape software. **(B)** The expression correlation between predicted miRNAs and CAPZA1 in LUAD as analyzed by the starBase database. **(C)** The expression correlation between miR-30d-5p and CAPZA1 in LUAD as analyzed by the starBase database. **(D)** The expression of miR-30d-5p in LUAD and control normal samples determined by the starBase database. **(E)** The prognostic value of let-7c-5p in LUAD assessed by the starBase database.

Noncoding RNAs (ncRNAs) are derived through the genome that fail to translate to proteins but regulate gene expression in cancers, including miRNAs and lncRNAs (29, 30). Thus, we aim to analyze the role of ncRNAs in regulating CAPZA1. Initially, we found 16 potential miRNAs that directly bond to CAPZA1 as its upstream (**Figures 4A, B**; **Supplementary Table 1**). As is well known, miRNAs could negatively modulate the expression of target genes by causing their cleavage or translational repression (31). As expected, CAPZA1 reversely correlated with hsa-miR-30a-5p, hsa-miR-30c-5p, hsa-miR-27b-3p, hsa-miR-488-3p, and hsa-miR-30d-5p in LUAD because of correlation analysis (**Figure 4B**, *P <*0.05). We then examined the expression and prognosis of the above miRNAs and found that only the hsa-miR-30d-5 in LUAD was at a low level, and its downregulation harbored a better life for patients (**Figures 4C–E**, *P <*0.05). These findings reveal that CAPZA1 might be the most potentially targeted mRNA of hsa-miR-30d-5 in LUAD.

### 3.4 Prediction and analysis of upstream lncRNAs of hsa-miR-30d-5p

**Figure 5:**
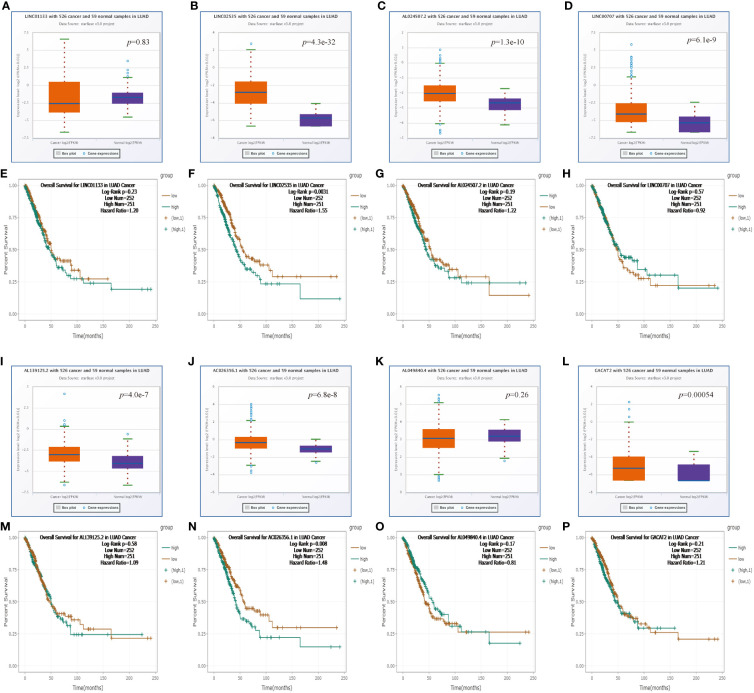
Expression analysis and survival analysis for upstream lncRNAs of miR-30d-5p in LUAD **(A–D, I–L)**. The expression of LINC01133 **(A)**, LINC02535 **(B)**, AL024507.2 **(C)**, LINC00707 **(D)**, AL139125.2 **(I)**, AC026356.1 **(J)**, AL049840.4 **(K)**, and GACAT2 **(L)** in the starBase database. **(E–H, M–P)** The OS analysis for LINC01133 **(E)**, LINC02535 **(F)**, AL024507.2 **(G)**, LINC00707 **(H)**, AL139125.2 **(M)**, AC026356.1 **(N)**, AL049840.4 **(O)**, and GACAT2 **(P)** in LUAD.

**Figure 6:**
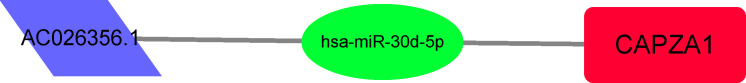
The model of AC026356.1–miR-30d-5p–CAPZA1 axis in LUAD.

Subsequently, we forecasted potential upstream lncRNAs of hsa-miR-30d-5p in LUAD through the starBase database and observed nine possible lncRNAs (**Supplementary Table 2**; **Supplementary Figure 2**). Based on the competitive endogenous RNA (ceRNA) hypothesis, LncRNA sponges various miRNAs to inhibit their expression and reduce the regulatory effect on target mRNA (32). Consequently, only AC026356.1 in LUAD showed higher activity than normal samples, and higher AC026356.1 was reciprocally linked to favorable patients’ OS among all the nine lncRNAs (**Figures 5A–P**). AC026356.1 lncRNA was positively relevant to CAPZA1 expression whereas negative with hsa-miR-30d-5p using the starBase database (**Tables 2**, **3**). To this end, AC026356.1 in LUAD might be most likely to interact with upstream lncRNAs of the hsa-miR-30d-5p-CAPZA1 axis (**Figure 6**).

**Table 2:** The correlation between lncRNA and CAPZA1 in lung adenocarcinoma.

LncRNA	GENE	R-value	*P*-value
LINC01133	CAPZA1	0.072	9.94E-02
LINC02535	CAPZA1	0.072	9.92E-02
AL024507.2	CAPZA1	0.218	4.27E-07
LINC00707	CAPZA1	0.034	4.38E-01
AL139125.2	CAPZA1	0.011	7.93E-01
AC026356.1	CAPZA1	0.317	1.01E-13
AL049840.4	CAPZA1	-0.129	3.14E-03
GACAT2	CAPZA1	0.24	2.52E-08

**Table 3:** The correlation between mir and lncRNA in lung adenocarcinoma.

miRNA	LncRNA	R-value	*P*-value
hsa-miR-30d-5p	LINC01133	-0.121	0.00595
hsa-miR-30d-5p	LINC02535	-0.128	0.00371
hsa-miR-30d-5p	AL024507.2	-0.293	1.29E-11
hsa-miR-30d-5p	LINC00707	-0.144	0.0011
hsa-miR-30d-5p	AL139125.2	-0.061	0.168
hsa-miR-30d-5p	AC026356.1	-0.299	4.71E-12
hsa-miR-30d-5p	AL049840.4	0.061	0.17
hsa-miR-30d-5p	GACAT2	-0.136	0.00198

### 3.5 CAPZA1-associated immune infiltration assessment in LUAD

**Figure 7:**
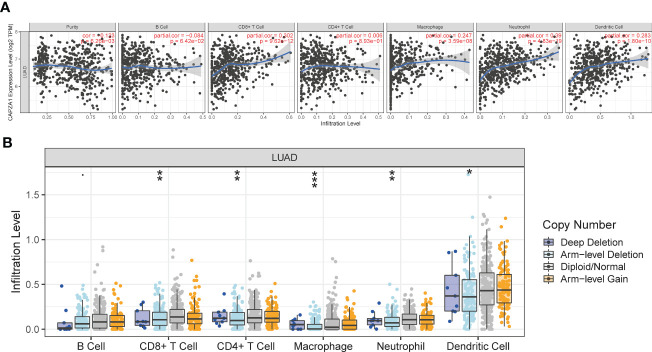
The relationship of immune cell infiltration with CAPZA1 level in LUAD. **(A)** The correlation of CAPZA1 expression level with B cell, CD8+ T cell, CD4+ T cell, macrophage, neutrophil, or dendritic cell infiltration level in LUAD. **(B)** The infiltration level of various immune cells under different copy numbers of CAPZA1 in LUAD. *p-value < 0.05; **p-value < 0.01; ***p-value < 0.001.

TME harbors comprehensively infiltrating immune cells, which contribute to influencing the development of cancers, so we investigated the relationship between CAPZA1 expression and immune infiltration. As presented in **Figure 7A**, the CAPZA1 expression level in LUAD was statistically positively related to macrophages, CD4+ T cells, CD8+ T cells, dendritic cells, and neutrophils, whereas the reciprocal association was with B cells and purity. Moreover, there was a significant change in immune cell infiltration under different copy numbers of CAPZA1 in LUAD (**Figure 7B**).

### 3.6 The link between CAPZA1 and immune checkpoints, IPS in LUAD

**Figure 8:**
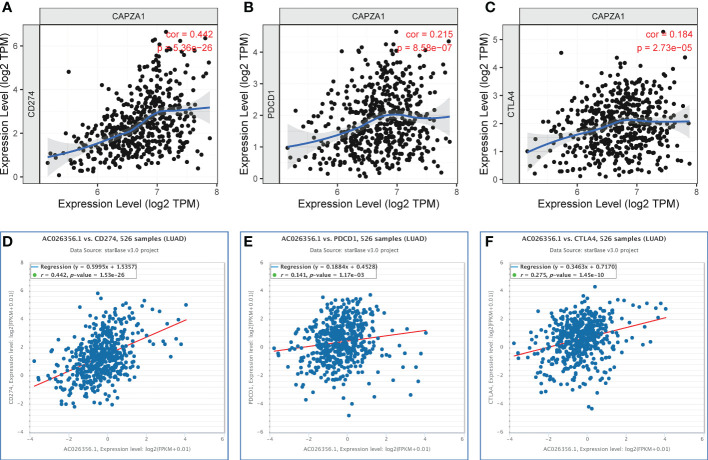
Correlation of CAPZA1 and AC026356.1 expression with PD-1, PD-L1, and CTLA-4 expression in LUAD. **(A)** Spearman correlation of CAPZA1 with expression of PD-L1 in LUAD using TIMER. **(B)** Spearman correlation of CAPZA1 with expression of PD-1 in LUAD using TIMER. **(C)** Spearman correlation of CAPZA1 with expression of CTLA-4 in LUAD using TIMER. **(D)** The expression correlation of AC026356.1 with PD-L1 in LUAD was determined by the starBase database. **(E)** The expression correlation of AC026356.1 with PD-1 in LUAD determined by the starBase database. **(F)** The expression correlation of AC026356.1 with CTLA-4 in LUAD was determined by the starBase database.

**Figure 9:**
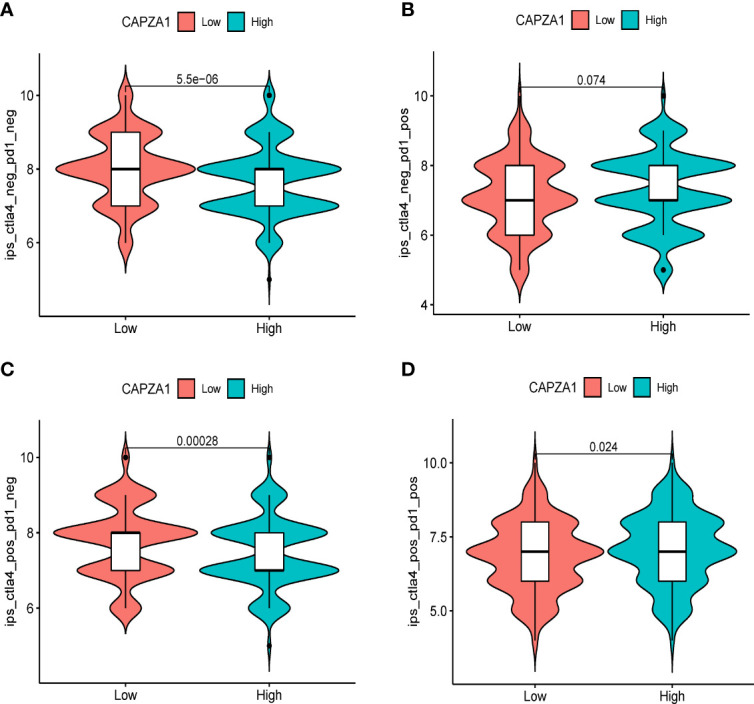
Correlation of CAPZA1 expression with the immunophenoscore (IPS) in HCC. **(A)** ips_ctla4_neg_pd1_neg, **(B)** ips_ctla4_neg_pd1_pos, **(C)** ips_ctla4_pos_pd1_neg, and **(D)** ips_ctla4_pos_pd1_pos.

PD1/PD-L1 and CTLA-4 are well-known immune checkpoints that play instrumental roles in cancer treatment modalities due to immune surveillance and escape. To ascertain the part of the immune response of CAPZA1 in LUAD with PD1, PD-L1, or CTLA-4, we utilized TIMER data to analyze the link between CAPZA1 and immune checkpoints. Indeed, high CAPZA1 and AC026356.1 expression was significantly positively correlated with PD1, PD-L1, and CTLA-4 in LUAD, respectively (**Figures 8A–F**), demonstrating that CAPZA1 can mediate tumorigenesis and progression by regulating tumor immune escape in LUAD. The IPS values (ips_ctla4_neg_pd1_pos, ips_ctla4_pos_pd1_neg, ips_ctla4_pos_pd1_neg, and ips_ctla4_pos_pd1_pos) have been reported in predicting cancer patients’ responses to anti-CTLA-4 treatment. Here, we noted a marked rise in scores in the low-CAPZA1 group (**Figures 9A–D**), implying that the low-CAPZA1 group is more suitable for anti-CTLA-4 and anti-PD-1/PD-L1 immunotherapy.

### 3.7 The correlation of CAPZA1 with immunotherapy and potential mechanisms

**Figure 10:**
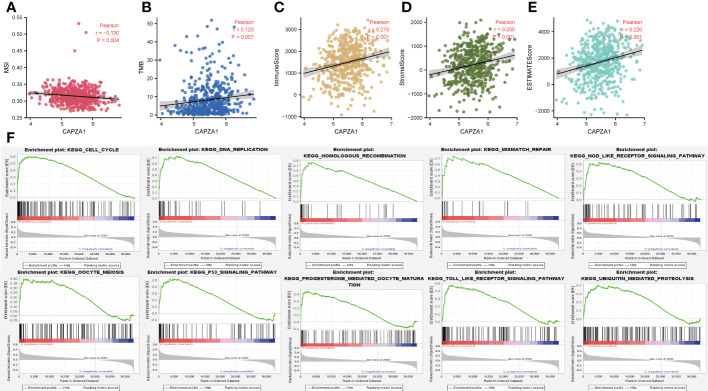
The correlation of CAPZA1 with immunotherapy and potential mechanisms. **(A)** Pearson correlation of CAPZA1 with MSI in LUAD. **(B)** Pearson correlation of CAPZA1 with TMB in LUAD. **(C)** Pearson correlation of CAPZA1 with ImmuneScore in LUAD. **(D)** Pearson correlation of CAPZA1 with StromalScore in LUAD. **(E)** Pearson correlation of CAPZA1 with estimateScores in LUAD. **(F)** Potential signaling pathway.

We further confirmed that CAPZA1 in LUAD was significantly positively correlated with the TMB, MSI, immune score, stromal score, and estimateScores (**Figure 10A–E**). GSEA revealed that CAPZA1 might be involved in cancer-related critical pathways such as cell cycle, P53 signal pathway, mismatch repair, Nod-like receptor signal pathway, and Toll-like receptor signal pathway (**Figure 10F**).

## 4 Discussion

LUAD is one of the significant invasive diseases limiting the life span of humans (33–35). Identifying the molecular mechanisms underlying LUAD carcinogenesis is becoming increasingly instrumental in searching for novel therapeutic targets and biomarkers. Previous studies have confirmed that CAPZA1 elicits pivotal roles in regulating the onset and development of human cancers (36, 37). However, the role of CAPZA1 in LUAD remains incompletely known and needs further study.

Our study first examined the finding that CAPZA1 was significantly overexpressed at mRNA levels in 13 tumors compared with normal tissues, which was validated in the starBase database. Moreover, survival and prognosis analysis displayed that higher CAPZA1 correlated with worse survival in KIRC, KIRP, LUAD, and LIHC using the starBase database. In addition, only the prognosis of CAPZA1 in LUAD had been validated in the PrognoScan and LOGpc databases, which might shed light on novel targets for LUAD individualized treatment.

Mechanisms related to ceRNA are modulated by ncRNAs with direct or indirect effects, including gene expression regulation (38). Our study used the starBase database to find possible miRNAs interacting with CAPZA1 RNA binding proteins, thereby modulating CAPZA1 mRNA stability. The result revealed that 16 miRNAs were finally found. Further analysis shows that only hsa-miR-30d-5p in LUAD was screened since it was low and its downregulation favored a better life for patients. Most of the research related to this miRNA has proved to be tumor-suppressive in many cancers, such as prostate cancer (39), renal cell carcinoma (RCC) (40), non-small cell lung cancer (NSCLC) (41), esophageal squamous cell carcinoma (ESCC) (42), and gallbladder (43). For instance, downregulated miR-30d-5p has been linked to favorable clinical outcomes in patients with RCC, and overexpression of miR-30d-5p attenuates RCC cell line proliferation and cell-cycle G1/S transition and facilitates apoptosis, which is denoted as a tumor-suppressor (40). CAPZA1 might be the most potentially targeted mRNA of hsa-miR-30d-5 in LUAD.

Recently, lncRNAs have been considered miRNA sponges that contribute to the regulation of cancers (44). Based on this, nine possible lncRNAs in LUAD function upstream of the hsa-miR-30d-5-CAPZA1 axis. Following survival analysis and correlation analysis, only AC026356.1 showed higher activity than normal samples, and higher AC026356.1 was reciprocally linked to favorable patients’ OS among all these lncRNAs. More importantly, AC026356.1 lncRNA was positively relevant to CAPZA1 expression whereas negative with hsa-miR-30d-5p, uncovering that AC026356.1 is most likely upstream lncRNAs of the hsa-miR-30d-5p-CAPZA1 axis.

Extensive evidence has proved that the tumor-infiltrating immune cells exert a crucial function on patients’ prognosis and immunotherapy (45). For example, activating tumor infiltrating CD8 T cells were able to contribute to antitumor responses (44). In our analysis, CAPZA1 in LUAD was positively relevant to the infiltration of most immune cells, especially macrophages, CD4+ T cells, CD8+ T cells, dendritic cells, and neutrophils, which is also validated based on some immune infiltration markers. Also, immunotherapy, mainly represented by immune checkpoint inhibitors (ICIs), has shown promising efficacy in treating many cancers (46). Thus, the linked immune checkpoints, including PD1, PD-L1, or CTLA-4 with CAPZA1, were assessed in LUAD, which has been successfully validated by IPS analysis, indicating that it might exert a pivotal role in restoring immune cytotoxic activity. Similarly, this correlation had the same trend, indicating that CAPZA1 might serve as a new tumor immunotherapy response predictor in LUAD. We further confirmed that CAPZA1 in LUAD was significantly positively correlated with the TMB, MSI, immune score, and stromal score, indicating that CAPZA1 could regulate immune infiltration function in LUAD. Moreover, GSEA revealed the tumor-associated and even tumor-promoting roles of CAPZA1, encompassing cancer-related critical pathways such as cell cycle, P53 signal pathway, mismatch repair, Nod-like receptor signal pathway, and Toll-like receptor signal pathway. It also displayed that CAPZA1 played a crucial role in tumor progression.

Nevertheless, some restrictions are supposed to be highlighted in our investigations. Firstly, considering tissue specificity, we fail to enable our conclusions to be reapplied to other tumors. Next, we need to validate the roles of CAPZA1 in LUAD on antitumor immunotherapy and tumor immunity based on more basic experiments. Finally, it is imperative to gather more robust data or samples for LUAD due to the lack of available public datasets.

For the first time, collectively, we comprehensively determine that CAPZA1 serves as an oncogene in pan-cancers and has a high CAPZA1 expression process that has an unfavorably high prognostic value in LUAD based on the starBase and PrognoScan databases. Our findings also uncovered that CAPZA1 might play an essential role in tumor immunity during the development of LUAD, such as immune infiltration cells, immune infiltration markers, TMB, MSI, immune score, stromal score, and immune checkpoints, and maybe a reliable therapeutic target for immunological antitumor strategies.

## Data availability statement

Publicly available datasets were analyzed in this study. This data can be found here: 1. https://xena.ucsc.edu/, 2. http://starbase.sysu.edu.cn/, 3. http://www.abren.net/PrognoScan/, 4. https://cistrome.shinyapps.io/timer/, 5. https://bioinfo.henu.edu.cn/DatabaseList.jsp.

## Author contributions

The project was designed and conceived by HPZ. HPZ analyzed the data. TQ and HYZ wrote the manuscript. WX and YM participated in the revision process, added analysis content, completed the picture layout, and answered relevant questions. ZY directed the revision process of the article and the revision of the article’s language. HPZ guided the execution of the study and revised the paper. All authors have read and approved the final version of this manuscript.

## Supplementary material

Supplementary Figure 1 Flow chart

Supplementary Figure 2 The lncRNAs of the miR-30d-5p regulatory network are established by cytoscape software.
