# Supplementary figures and images for: NcRNA-regulated CAPZA1 associated with prognostic and immunological effects across lung adenocarcinoma
Source: Front Oncol. 2023 Jan 4;12:1025192. doi: 10.3389/fonc.2022.1025192 (PMC9846042; doi:10.3389/fonc.2022.1025192)

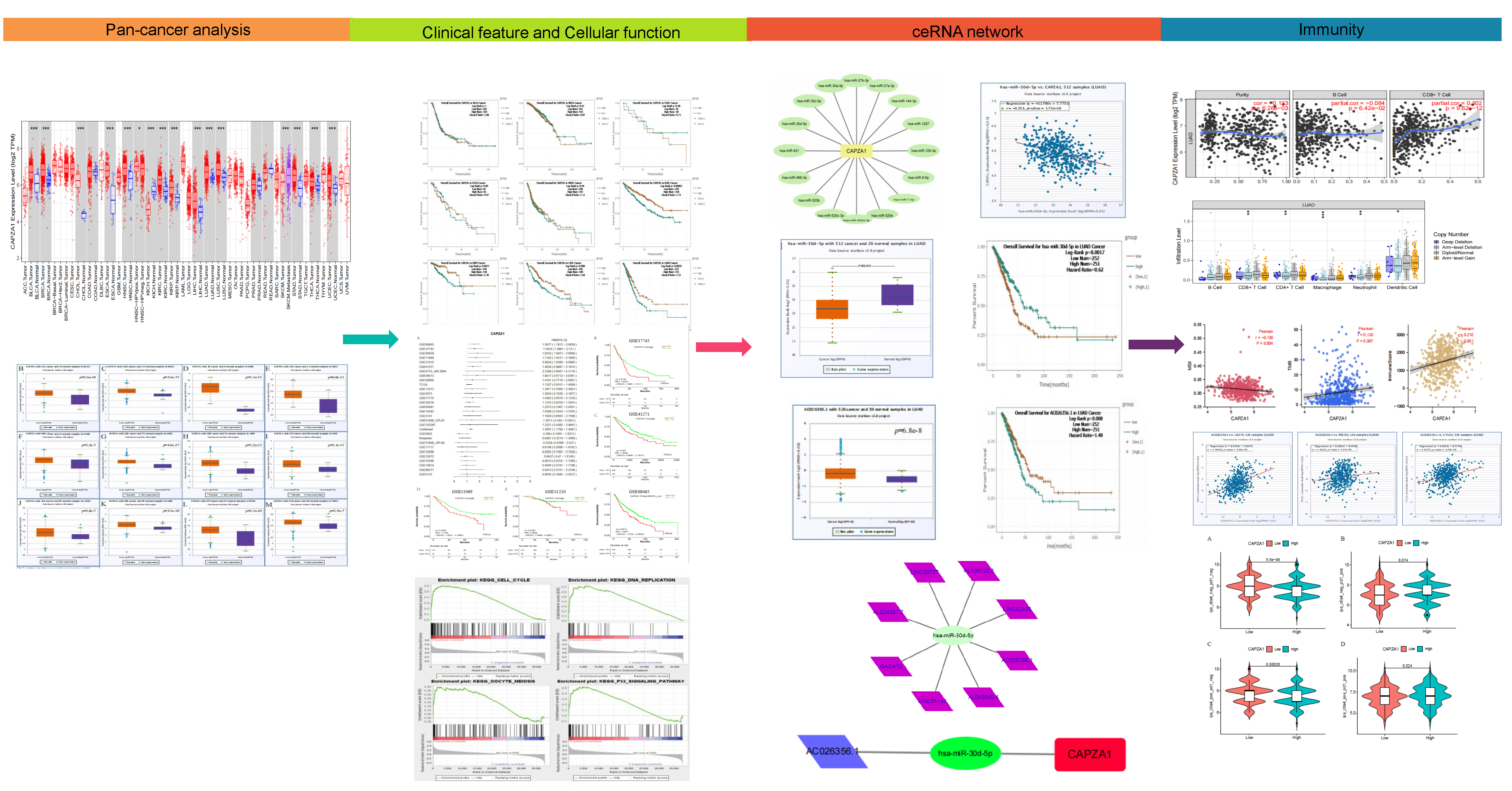

Supplement: Supplementary Figure 1 — Flow chart [file Image_1.tif]

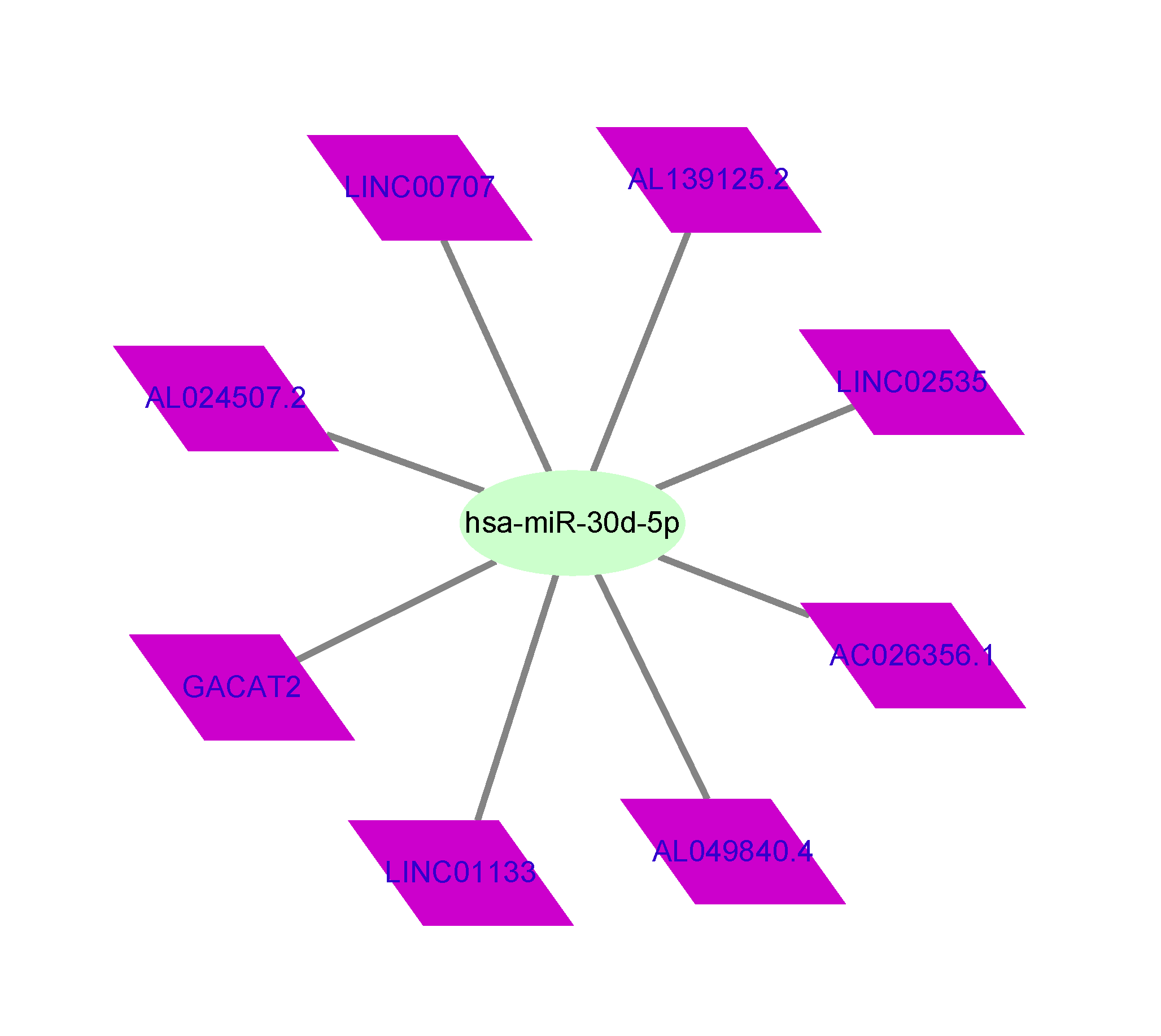

Supplement: Supplementary Figure 2 — The lncRNAs of the miR-30d-5p regulatory network are established by cytoscape software. [file Image_2.tif]
