# Supplementary material for: NcRNA-regulated CAPZA1 associated with prognostic and immunological effects across lung adenocarcinoma
Source: Front Oncol. 2023 Jan 4;12:1025192. doi: 10.3389/fonc.2022.1025192 (PMC9846042; doi:10.3389/fonc.2022.1025192)
Supplement: Supplementary file 3 [file Table_1.docx]

| **Supplementary Table 1.** The upstream miRNAs of CAPZA1 from starbase. | | | | | | | | | | | | | | | | | | | | | |
| --- | --- | --- | --- | --- | --- | --- | --- | --- | --- | --- | --- | --- | --- | --- | --- | --- | --- | --- | --- | --- | --- |
| miRNAid | miRNAname | geneID | geneName | geneType | chromosome | narrowStart | narrowEnd | broadStart | broadEnd | strand | clipExpNum | degraExpNum | RBP | PITA | RNA22 | miRmap | microT | miRanda | PicTar | TargetScan | pancancerNum |
| MIMAT0000084 | hsa-miR-27a-3p | ENSG00000116489 | CAPZA1 | protein_coding | chr1 | 1.13E+08 | 1.13E+08 | 1.13E+08 | 1.13E+08 | + | 15 | 0 | AGO1,AGO1-4,AGO2,AGO3 | 1 | 0 | 1 | 1 | 1 | 1 | 1 | 1 |
| MIMAT0000087 | hsa-miR-30a-5p | ENSG00000116489 | CAPZA1 | protein_coding | chr1 | 1.13E+08 | 1.13E+08 | 1.13E+08 | 1.13E+08 | + | 30 | 0 | AGO1,AGO1-4,AGO2,AGO3 | 1 | 0 | 0 | 1 | 1 | 1 | 1 | 15 |
| MIMAT0000244 | hsa-miR-30c-5p | ENSG00000116489 | CAPZA1 | protein coding | chr1 | 1.13E+08 | 1.13E+08 | 1.13E+08 | 1.13E+08 | + | 30 | 0 | AGO1,AGO1-4,AGO2,AGO3 | 1 | 0 | 0 | 1 | 1 | 1 | 1 | 12 |
| MIMAT0000245 | hsa-miR-30d-5p | ENSG00000116489 | CAPZA1 | protein coding | chr1 | 1.13E+08 | 1.13E+08 | 1.13E+08 | 1.13E+08 | + | 30 | 0 | AGO1,AGO1-4,AGO2,AGO3 | 1 | 0 | 0 | 1 | 1 | 1 | 1 | 15 |
| MIMAT0000252 | hsa-miR-7-5p | ENSG00000116489 | CAPZA1 | protein coding | chr1 | 1.13E+08 | 1.13E+08 | 1.13E+08 | 1.13E+08 | + | 10 | 0 | AGO1-4,AGO2 | 1 | 0 | 1 | 1 | 1 | 1 | 1 | 4 |
| MIMAT0000419 | hsa-miR-27b-3p | ENSG00000116489 | CAPZA1 | protein coding | chr1 | 1.13E+08 | 1.13E+08 | 1.13E+08 | 1.13E+08 | + | 15 | 0 | AGO1,AGO1-4,AGO2,AGO3 | 1 | 0 | 1 | 1 | 1 | 1 | 1 | 13 |
| MIMAT0000424 | hsa-miR-128-3p | ENSG00000116489 | CAPZA1 | protein_coding | chr1 | 1.13E+08 | 1.13E+08 | 1.13E+08 | 1.13E+08 | + | 14 | 0 | AGO1,AGO1-4,AGO2 | 1 | 0 | 0 | 1 | 1 | 1 | 1 | 5 |
| MIMAT0000436 | hsa-miR-144-3p | ENSG00000116489 | CAPZA1 | protein_coding | chr1 | 1.13E+08 | 1.13E+08 | 1.13E+08 | 1.13E+08 | + | 14 | 0 | AGO1,AGO1-4,AGO2 | 1 | 0 | 1 | 1 | 1 | 1 | 1 | 0 |
| MIMAT0000441 | hsa-miR-9-5p | ENSG00000116489 | CAPZA1 | protein_coding | chr1 | 1.13E+08 | 1.13E+08 | 1.13E+08 | 1.13E+08 | + | 18 | 0 | AGO1,AGO1-4,AGO2 | 1 | 0 | 1 | 1 | 0 | 1 | 1 | 7 |
| MIMAT0002825 | hsa-miR-520e | ENSG00000116489 | CAPZA1 | protein_coding | chr1 | 1.13E+08 | 1.13E+08 | 1.13E+08 | 1.13E+08 | + | 23 | 0 | AGO1,AGO1-4,AGO2 | 1 | 0 | 1 | 1 | 1 | 0 | 1 | 0 |
| MIMAT0002843 | hsa-miR-520b | ENSG00000116489 | CAPZA1 | protein_coding | chr1 | 1.13E+08 | 1.13E+08 | 1.13E+08 | 1.13E+08 | + | 23 | 0 | AGO1,AGO1-4,AGO2 | 1 | 0 | 1 | 1 | 1 | 0 | 1 | 0 |
| MIMAT0002846 | hsa-miR-520c-3p | ENSG00000116489 | CAPZA1 | protein_coding | chr1 | 1.13E+08 | 1.13E+08 | 1.13E+08 | 1.13E+08 | + | 23 | 0 | AGO1,AGO1-4,AGO2 | 1 | 0 | 1 | 1 | 1 | 0 | 1 | 0 |
| MIMAT0002856 | hsa-miR-520d-3p | ENSG00000116489 | CAPZA1 | protein_coding | chr1 | 1.13E+08 | 1.13E+08 | 1.13E+08 | 1.13E+08 | + | 23 | 0 | AGO1,AGO1-4,AGO2 | 1 | 0 | 1 | 1 | 1 | 0 | 1 | 0 |
| MIMAT0003339 | hsa-miR-421 | ENSG00000116489 | CAPZA1 | protein_coding | chr1 | 1.13E+08 | 1.13E+08 | 1.13E+08 | 1.13E+08 | + | 14 | 0 | AGO1,AGO1-4,AGO2 | 1 | 0 | 0 | 1 | 1 | 1 | 1 | 0 |
| MIMAT0004763 | hsa-miR-488-3p | ENSG00000116489 | CAPZA1 | protein_coding | chr1 | 1.13E+08 | 1.13E+08 | 1.13E+08 | 1.13E+08 | + | 12 | 0 | AGO1,AGO1-4,AGO2 | 1 | 0 | 1 | 1 | 1 | 0 | 1 | 11 |
| MIMAT0005886 | hsa-miR-1297 | ENSG00000116489 | CAPZA1 | protein_coding | chr1 | 1.13E+08 | 1.13E+08 | 1.13E+08 | 1.13E+08 | + | 7 | 0 | AGO1-4,AGO2 | 1 | 0 | 0 | 1 | 1 | 1 | 1 | 1 |
