# Supplementary material for: NcRNA-regulated CAPZA1 associated with prognostic and immunological effects across lung adenocarcinoma
Source: Front Oncol. 2023 Jan 4;12:1025192. doi: 10.3389/fonc.2022.1025192 (PMC9846042; doi:10.3389/fonc.2022.1025192)
Supplement: Supplementary file 4 [file Table_2.docx]

| **Supplementary Table 2.** miRNA-target lncRNA of hsa-miR-30d-5p from starbase. | | | | | | | | | | | | | | | | |
| --- | --- | --- | --- | --- | --- | --- | --- | --- | --- | --- | --- | --- | --- | --- | --- | --- |
| miRNAid | miRNAname | geneID | geneName | geneType | chromosome | start | end | strand | clipExpNum | degraExpNum | RBP | merClass | miRseq | align | targetSeq | pancancerNum |
| MIMAT0000245 | hsa-miR-30d-5p | ENSG00000224259 | LINC01133 | lincRNA | chr1 | 1.6E+08 | 1.6E+08 | + | 1 | 0 | AGO1-4 | 7mer-m8 | gaaggucaGCCCCUACAAAUGu | :\| \| \|\|\|\|\|\|\| | agugccucUGAGUUUGUUUACu | 12 |
| MIMAT0000245 | hsa-miR-30d-5p | ENSG00000234155 | LINC02535 | lincRNA | chr6 | 86099212 | 86099231 | - | 1 | 0 | AGO1-4 | 7mer-m8 | gaAGGUCAGCCCCUACAAAUGu | \|:\|\|\| \| \| \|\|\|\|\|\|\| | ccUUCAGACAAG--UGUUUACu | 12 |
| MIMAT0000245 | hsa-miR-30d-5p | ENSG00000272476 | AL024507.2 | antisense | chr6 | 1.08E+08 | 1.08E+08 | + | 1 | 0 | AGO1-4 | 7mer-m8 | gaaggucagccccUACAAAUGu | \|\|\|\|\|\|\|\| | uguuuucaaagcaAUGUUUACu | 12 |
| MIMAT0000245 | hsa-miR-30d-5p | ENSG00000238266 | LINC00707 | lincRNA | chr10 | 6883163 | 6883184 | + | 2 | 0 | AGO2 | 7mer-m8 | gaaGGUCAGCCCCUACAAAUGu | ::\| \|\| \| \|\|\|\|\|\|\| | uuaUUAAUCUAUGUUGUUUACu | 10 |
| MIMAT0000245 | hsa-miR-30d-5p | ENSG00000278982 | AL139125.2 | TEC | chr10 | 7455339 | 7455360 | + | 1 | 0 | AGO1-4 | 7mer-m8 | gaAGGUCAGCCCCUACAAAUGu | \|\|: \| \|\| \|\|\|\|\|\|\| | gcUCUCUCCAGGCUUGUUUACc | 12 |
| MIMAT0000245 | hsa-miR-30d-5p | ENSG00000274964 | AC026356.1 | sense_intronic | chr12 | 32493560 | 32493581 | + | 2 | 0 | AGO1-4,AGO2 | 8mer | gaaggucagccccuACAAAUGu | \|\|\|\|\|\|\| | guaugacugguuuuUGUUUACa | 11 |
| MIMAT0000245 | hsa-miR-30d-5p | ENSG00000269958 | AL049840.4 | sense_intronic | chr14 | 1.04E+08 | 1.04E+08 | + | 5 | 0 | AGO1-4 | 8mer | gaaggucagccccUACAAAUGu | :\|\|\|\|\|\|\| | #NAME? | 13 |
| MIMAT0000245 | hsa-miR-30d-5p | ENSG00000269958 | AL049840.4 | sense_intronic | chr14 | 1.04E+08 | 1.04E+08 | + | 4 | 0 | AGO1-4,AGO2 | 8mer | gaAGGUCAGCCCCU---ACAAAUGu | \| :\|\| \|\| \| \| \|\|\|\|\|\|\| | uaUGUAGACGUGUAUUCUGUUUACa | 13 |
| MIMAT0000245 | hsa-miR-30d-5p | ENSG00000265962 | GACAT2 | antisense | chr18 | 8695940 | 8695961 | - | 2 | 0 | AGO1-4 | 8mer | gaaggucagCCCCUACAAAUGu | \|\|\| \|\|\|\|\|\|\|\| | acaaaauaaGGGUAUGUUUACa | 11 |
